# Gaps in sexually transmitted infection screening among youth living with HIV in Alabama

**DOI:** 10.1186/s13104-022-06241-7

**Published:** 2022-11-08

**Authors:** Henna Budhwani, Jiaying Hao, Allysha C. Maragh-Bass, Samantha V. Hill, Dustin M. Long, Tina Simpson

**Affiliations:** 1 College of Nursing, Florida State University, Tallahassee, FL USA; 2 School of Public Health, University of Alabama at Birmingham, Birmingham, AL USA; 3 Behavioral, Epidemiological, and Clinical Sciences Division, FHI 360 Durham, NC USA; 4 Duke Global Health Institute, Duke University, Durham, NC USA; 5 School of Medicine, University of Alabama at Birmingham, Birmingham, AL USA

**Keywords:** Youth, HIV, Sexually transmitted infections, Gonorrhea, Chlamydia, Syphilis, Alabama, Testing

## Abstract

**Objective:**

Gaps in sexually transmitted infection (STI) testing can lead to poor health outcomes due to untreated illness among youth living with HIV (YLHIV). Thus, the objective of this study is to examine STI testing behavior and outcomes among a sample of YLHIV in the southern United States. Clinical records of 139 YLHIV who received HIV care in Alabama (2017–2020) were evaluated for receipt of STI testing (gonorrhea, chlamydia, syphilis), prevalence of positive test results, and factors associated with testing outcomes (933 clinical visits).

**Results:**

Nearly 80% of our sample identified as African American, most were 20–24 years, and about 60% reported detectable viral load at first visit during the study period. Just under 60% of cisgender male and transgender female clients reported receipt of at least one STI test, compared to less than 40% of cisgender females. Identifying as a cisgender male and having been diagnosed with HIV related to sex with men were associated with greater likelihood receiving STI testing. Cisgender males reported higher rates of positive syphilis test results than cisgender females; the highest rates of positive STI tests were among transgender females. Results underscore need for providers to promote routine STI testing to YLHIV.

## Introduction

Sexually transmitted infections (STIs) are a common co-morbidity experienced by youth living with HIV (YLHIV) [1]. Clinical guidelines recommend people living with HIV (PLHIV) receive chlamydia, gonorrhea, and syphilis screening at least annually, and more frequent screening is recommended for clients who may have multiple sexual partners [2]. Youth are over-represented among new HIV cases, and YLHIV are at higher risk of STI infection as compared to older populations who have been living with HIV for more years [1, 3]. This is of particular note, because STIs among YLHIV can negatively impact their HIV-specific health outcomes [4]. Beyond challenges in managing HIV, the treatment of STIs and PLHIV may be more complex due to immunocompromised status. More research is needed to understand correlates of STI prevalence and preventative behaviors among YLHIV to inform STI testing interventions.

Adherence to regular screening STI screening protocols for PLHIV is crucial to the Health and Human Resource Service Administration’s (HRSA) goal of reducing new HIV infections by 90% by 2030 as stated in their Plan for Ending the HIV Epidemic (EHE) [5]. As the social networks of YLHIV grow increasingly complex, regular STI screening provides a mechanism to link sero-discordant partners to prevention and treatment services in a timelier fashion and is aligned with recommended treatment as prevention approaches (TasP) [6]. Thus, STI testing of PLHIV and linkage of their sexual partners to STI services, contributes to the diagnosis pillar of HRSA’s EHE [5]. Studies suggest that routine STI testing among PLHIV is inconsistent and may be reducing [7]. Therefore, the purpose of this exploratory study is to examine STI screening behaviors and testing outcomes among southern YLHIV.

## Methods

### Data and sample

The study sample includes a sample of YLHIV aged 10–24 years from a HRSA-funded Ryan White Part B and D clinic in Alabama (N = 139). Data were extracted from multiple electronic medical records systems. Patient profiles were developed through aggregations of 933 clinical visits from 2017 to 2020. The first clinical record was denoted as an index visit, indicating the point at which the youth entered into care or the first record of an established patient during the timeframe.

### Ethics review

Study materials were reviewed and approved by the University of Alabama at Birmingham Institutional Review Board.

### Measures: demographics

Gender, age, and race were extracted from the index visit record. Age ranged from 10 to 24 years. Gender categories were cisgender female, cisgender male, and transgender female. No records indicated gender of transgender male. Race was coded as African American, White, and Other. We did not include ethnicity due to small sample size (Hispanic, n = 3).

### Measures: STI testing

STI testing measures were identified by ICD-10 codes. We created individual STI testing measures for chlamydia, gonorrhea, and syphilis. Each measure included a response indicating if a test was performed for each infection (Yes = 1; No Test = 0). For records that included an STI test, we noted results. A reactive or positive test was coded as 1 (Negative = 0). We then created a variable that was an aggregation of test or no test, named Presence of Testing. For Presence of Testing, if any STI tests were in the patient’s health records the variable was coded = 1; no tests was coded = 0. Finally, we created an aggregated STI outcome measure. If the record included any reactive test result (chlamydia, gonorrhea, or syphilis), this variable was coded = 1 for positive screen. If all test results were non-reactive or negative, the variable was coded = 0.

### Measures: HIV Mode of transmission

Mode of HIV transmission was coded: mother-to-child transmission (coded = perinatal), through sexual intercourse between men who have sex with men (MSM, coded = anal sex), and other types of transmission, including but not limited to intravenous drug use (coded = other).

### Statistical analyses

To investigate relationships across outcomes, we applied univariate generalized linear mixed models with generalized logit link. Comparisons were made with negative test result as referent. Random intercepts for each client were considered to control for within person variability. Analyses were performed with SAS (Version 9.4, Cary, North Carolina), with PROC GLIMMIX procedure approximating the marginal likelihood by using Laplace’s method. The empirical estimators were applied as the computational option to increase robustness. Unadjusted odds ratios (OR), 95% confidence intervals (CI) and p-values (p) are reported.

## Results

### Sample characteristics

Median age was 22 years (interquartile range: 19–23 years). Over half were 20–24 years old (69.1%), cisgender male (67.6%), and African American (77.0%). Sex among MSM was the most common route of acquisition (54.7%) following by perinatal transmission (25.2%).

#### STI testing by gender

For cisgender males, 565 visits were evaluated. Among visits, 317 denoted at least one STI test (56.1%). Records included 259 chlamydia, 258 gonorrhea, and 202 syphilis tests; 24 chlamydia (9.3%), 17 gonorrhea (6.6%) and 74 syphilis (36.6%) cases were confirmed. Cisgender female YLHIV had 356 visits. Among these 356 visits, 139 were recorded with at least one STI test (39.0%). Of these tests, 119 were for chlamydia, 118 for gonorrhea, and 81 for syphilis; 8 chlamydia (2.2%), 4 gonorrhea (3.4%) and 5 syphilis cases were confirmed (6.2%). Twelve visits were recorded among transgender female patients; 7 visits included at least one STI test (58.3%). Two chlamydia (33.3%) and 2 syphilis (66.6%) cases were confirmed. See Table 1.

**Table 1:** Gender by Sexually Transmitted Infection Laboratory Test Result

Gender	# Visits	Chlamydia		Gonorrhea		Syphilis		STI Test (Yes)
		Test	Positive	Test	Positive	Test	Positive	
Cisgender male	565	259	24	258	17	202	74	317
Cisgender female	356	119	8	118	4	81	5	139
Transgender female	12	6	2	6	0	3	2	7

### STI results by testing

Cisgender males had higher odds of receiving STI testing than cisgender females; chlamydia (OR = 1.71, CI:1.16, 2.51); gonorrhea (OR = 1.71, CI:1.17, 2.50), and syphilis (OR = 1.89, CI:1.43, 2.49). MSM had increased odds of having all types of tests than clients who contracted HIV through others modes of transmission: chlamydia (OR = 1.87, CI:1.25, 2.81), gonorrhea (OR = 1.91, CI:1.29, 2.81), and syphilis (OR = 2.12, CI:1.63, 2.76). Clients who contracted HIV perinatally had reduced odds of testing for chlamydia (OR = 0.55, CI:0.31, 0.98) and gonorrhea (OR = 0.55, CI:0.31, 0.96) compared to clients who acquired HIV via other modes of transmission. See Table 2.

**Table 2:** Unadjusted Tests of STI test and STI Result

Effects		Chlamydia			Gonorrhea			Syphilis		
		OR	CI	p	OR	CI	p	OR	CI	p
Gender										
Test vs. No test	Transgender vs. Cisgender Female	2.47	(0.65, 9.43)	0.0183	2.45	(0.66, 9.09)	0.0144	1.13	(0.59, 2.17)	< 0.0001
	Cisgender Male vs. Cisgender Female	1.71	(1.16, 2.51)		1.71	(1.17, 2.50)		1.89	(1.43, 2.49)	
no test vs. Negative	Transgender vs. Cisgender Female	0.48	(0.12, 1.84)	< 0.0001	0.28	(0.06, 1.32)	< 0.0001	2.01	(0.18, 23.89)	0.003
	Cisgender Male vs. Cisgender Female	0.60	(0.39, 0.94)		0.61	(0.39, 0.94)		0.95	(0.68, 1.32)	
Positive vs. Negative	Transgender vs. Cisgender Female	23.13	(5.06, 105.77)		0.01	(0.00, 0.07)		0	(0.00, 264,640)	
	Cisgender Male vs. Cisgender Female	1.9	(0.62, 5.82)		1.57	(0.57, 4.37)		25.49	(3.02, 215.30)	
Race										
Test vs. No test	White vs. African American	0.73	(0.47, 1.12)	0.0897	0.74	(0.48, 1.13)	0.1001	0.37	(0.54, 1.26)	0.5832
	Other vs. African American	0.36	(0.12, 1.09)		0.36	(0.12, 1.11)		0.55	(0.30, 1.89)	
no test vs. Negative	White vs. African American	1.28	(0.83, 1.98)	0.0174	1.34	(0.88, 2.03)	0.1643	0.93	(0.63, 1.37)	0.0914
	Other vs. African American	2.88	(0.96, 8.61)		2.95	(1.00, 8.71)		1.71	(0.73, 3.96)	
Positive vs. Negative	White vs. African American	0.31	(0.07, 1.48)		0.77	(1.75, 3.42)		0.12	(0.02, 0.75)	
	Other vs. African American	1.45	(0.75, 2.81)		2.36	(0.40, 14.09)		1.48	(0.15, 14.73)	
HIV Mode of Transmission										
Test vs. No test	MSM via Anal Sex vs. Other	1.87	(1.25, 2.81)	< 0.0001	1.91	(1.29, 2.81)	< 0.0001	2.12	(1.63, 2.76)	< 0.0001
	Perinatal vs. Other	0.55	(0.31, 0.98)		0.55	(0.31, 0.96)		0.86	(0.56, 1.30)	
no test vs. Negative	MSM via Anal Sex vs. Other	0.51	(0.32, 0.81)	< 0.0001	0.51	(0.33, 0.79)	< 0.0001	0.87	(0.62, 1.22)	< 0.0001
	Perinatal vs. Other	1.72	(0.91, 3.27)		1.73	(0.93, 3.25)		1.00	(0.61, 1.54)	
Positive vs. Negative	MSM via Anal Sex vs. Other	2.82	(0.62, 12.81)		1.63	(0.54, 4.94)		25.68	(3.44, 191.76)	
	Perinatal vs. Other	1.54	(0.27, 8.68)		0.49	(0.06, 4.09)		0	(0.00, 0.00)	

### STI test results

Cisgender males had 40% lower odds of not testing rather than receiving a negative result compared to cisgender females (OR = 0.6; CI = 0.39, 0.94). Transgender females had higher odds of having a positive chlamydia result compared to cisgender females (OR = 23.13; CI = 5.06, 105.77). MSM had notably lower odds of having no test rather than a negative test result compared to youth who contracted HIV via other modes (OR = 0.51, CI:0.32, 0.81). White YLHIV had higher odds of having a negative chlamydia result than a positive result than other racial identities (OR = 5.00, CI:1.16, 21.54). For gonorrhea results, gender (p < 0.01) and HIV mode of transmission (p < 0.01) were statistically significant. Transgender females had 99% lower odds of having a positive gonorrhea test than a negative result compared to cisgender females (OR = 0.01, CI:0.00, 0.07), and cisgender males had lower odds of having no gonorrhea test than a negative result as compared to cisgender females (OR = 0.61, CI:0.39, 0.94). YLHIV who contracted HIV through MSM sexual behaviors had lower odds of having no gonorrhea test than a negative result compared to those who contracted HIV through other modes of transmission (OR = 0.51, CI:0.33, 0.79). Cisgender males had higher odds of having positive syphilis test results compared to cisgender females (OR = 25.49, CI:3.02, 215.30). MSM had higher odds of positive syphilis result compared to YLHIV who contracted HIV through other modes of transmission (OR = 25.68, CI:3.44, 191.76). Perinatally-infected YLHIV had the lowest odds of positive syphilis result versus a negative result (p < 0.01). See Table 2.

## Discussion

We found adherence to STI clinical testing guidelines was low. Results underscore the need to evaluate structural, clinical, and personal barriers to STI testing among YLHIV. Regression results indicated multiple factors were associated with receipt of any STI test whereby gender and HIV mode of transmission were significant. Cisgender males were far more likely than cisgender females to have received an STI test and were more likely to receive positive STI results. Gender and mode of transmission were both associated with receipt of chlamydia tests and gonorrhea tests. The majority of our sample were MSM underscoring the potential benefit for creation of interventions to increase routine STI testing among MSM and MSM YLHIV [8]. Clients were predominantly African American, and the clinic is located in Alabama where some of the greatest HIV inequities and structural barriers are experienced.

### Future research

Results support the need for further exploration of healthcare and structural factors’ influence on the sexual health of YLHIV in the southern United States. Despite our small sample size, results lend credence for warranted exploration of predictors of STI testing and treatment among MSM and transgender female YLHIV who continue to experience the brunt of HIV inequities. Results support the need for additional research on STI testing among YLHIV as part of clinical protocols implemented in order to make meaningful progress towards ending the epidemic.

### Limitations

Data were cross-sectional, preventing ascertainment of causal direction of associations among the variables. Although sexual risk assessment informs testing (if a client states he is sexually inactive, the provider may not offer testing), we were unable to capture this measure. “Male” and “men” were considered equivalent both theoretically and analytically, which may be simplistic given that sex is biological while gender is sociocultural. Our sample only included YLHIV in a single clinic in Alabama, limiting generalizability.

## Data Availability

The administrative datasets generated and analysed during the current study are not publicly available due to the datasets including personal health information from highly vulnerable pediatric populations but are available from the senior author on reasonable request.
